# Fixation stability after surgical treatment of strabismus and biofeedback fixation training in amblyopic eyes

**DOI:** 10.1186/s12886-021-02020-3

**Published:** 2021-06-24

**Authors:** Otto Alexander Maneschg, Mirella Telles Salgueiro Barboni, Zoltán Zsolt Nagy, János Németh

**Affiliations:** 1 Department of Ophthalmology, Semmelweis University, Budapest, Hungary; 2 Bionic Innovation Center, Budapest, Hungary

**Keywords:** Strabismus, Amblyopia, Microperimetry, Biofeedback fixation training, Squint surgery

## Abstract

**Background:**

Visual fixation may be affected in amblyopic patients and, moreover, its stability may be associated with the effects of amblyopic treatments on visual performance in patients with strabismus. Therefore, fixation stability is a relevant biomarker that might predict the recurrence of amblyopia after a therapeutic intervention. Microperimetric biofeedback fixation training (BFT) can stabilize visual fixation in adult patients with central vision loss. It was the purpose of the present study to evaluate the effects of BFT on fixation stability in adult amblyopic patients after surgical intervention to treat strabismus.

**Methods:**

Participants were 12 patients with strabismus (mean age = 29.6 ± 8.5 years; 6 females) and 12 healthy volunteers (mean age = 23.8 ± 1.5 years; 9 females). The protocol included ophthalmological and microperimetric follow-ups to measure fixation stability and macular sensitivity. BFT was applied monocularly to four amblyopic eyes either on the spontaneous preferential retinal locus or to a fixation area closer to the anatomical fovea after surgical treatment of strabismus.

**Results:**

Baseline measurements showed significantly altered microperimetric average threshold in amblyopic eyes compared to fellow eyes (*p* = 0.024) and compared to control eyes (*p* < 0.001). Fixation was unstable in amblyopic eyes compared to control eyes (p < 0.001). Fixation stability did not significantly change after surgical alignment of strabismus (*p* = 0.805). BFT applied to operated eyes resulted in a more stable fixation with improvements of about 50% after three months of training.

**Conclusions:**

Fixation stability improvements following BFT highlight its potential use in adult amblyopic eyes after the surgical alignment of the strabismus. Future investigations may also consider applying this method in combination with standard treatments to improve vision in amblyopic patients.

**Supplementary Information:**

The online version contains supplementary material available at 10.1186/s12886-021-02020-3.

## Background

Amblyopia is characterized by reduced visual acuity [1] associated with structural brain [2, 3] and retinal [4] changes that are usually not observed during the standard ophthalmological examination. It occurs due to asymmetric interocular suppression of visual inputs to the cortex, very often caused by the misalignment (strabismus) of the eyes [5–7] and / or unequal and uncorrected refractive error (anisometropia) [8], and stimulus deprivation due to an obstruction of visual pathway [7, 9]. In amblyopic adult eyes, cosmetic results are believed to be the main benefit for patients with chronic strabismus undergoing surgical treatment. Nevertheless, several studies have proved that binocular summation can be modulated after the alignment of the eyes [10, 11] and elimination of diplopia and/or a compensatory head posture [12–14]. However, the surgical alignment of the strabismic eyes in adult with amblyopia is usually not followed by visual acuity improvements. Moreover, it has been associated with risk of squint relapse [15].

Fixation stability is often affected in amblyopic patients [16]. It has been recently reported that visual acuity improvements are accompanied by changes in fixation stability after amblyopic treatment [17]. Therefore, fixational eye movements may be considered in amblyopic treatment. Microperimetry, or fundus-controlled visual field examination, is a relatively new method to measure monocular macular light sensitivity while recording eye movements to monitor visual fixation (for review see Rohrschneider et al., 2008) [18]. In addition, the continuous recording of eye movements during the examination enables the establishment of the preferred retinal locus (PRLs), which is the preferential location on which the tested eye fixates a central target. This approach allows recording and quantifying fixation stability [19].

The biofeedback fixation training (BFT), integrated in some microperimetric systems, allows selecting one retinal location to be stimulated for either providing a more stable fixation or to change PRL retinal location [20, 21]. It has been earlier reported [22, 23] and increasingly emphasized [24–27] that BFT improves fixation stability in patients with low vision. The conditions causing low vision due to retinal alterations in macular diseases may stimulate the replacement of the fixation area to a less affected surrounding area (eccentric viewing). In contrast, amblyopic patients with strabismus may display eccentric fixation, in which the visual center (functional fovea) is shifted to a non-foveal area due to suppressive mechanisms during the critical period of development [28]. It was long demonstrated that visual fixation can be improved with auditory feedback in amblyopia [29]. More recently, functional gain was demonstrated in children and adults undergoing visual training using pattern stimulation associated with auditory feedback [30, 31].

We hypothesize that BFT providing a better control of microsaccades could improve fixation stability in amblyopic adult eyes after surgical alignment of the strabismus. The purpose of the present study was to investigate the effects of surgical treatment and BFT on fixation stability.

## Methods

### Patients

The study was performed according to the tenets of the Declaration of Helsinki and was approved by the ethics committee of the National Healthcare Service Center (Egészségügyi Nyilvántartási és Képzési Központ, registration number ENKK 037871–006/2016/OTIG) and by the National Institute of Pharmacy and Nutrition (Országos Gyógyszerészeti és Élelmezés-egészségügyi Intézet, registration number OGYÉI/42821/2019). All participants were informed about the nature and possible consequences of the study giving their written consent to participate in the study.

Participants were 12 patients with strabismus (mean age = 29.6 ± 8.5 years; 6 females; 9 amblyopic) and 12 healthy volunteers (mean age = 23.8 ± 1.5 years; 9 females). Table 1 shows pre / postoperative clinical data from the patients recorded before the BFT. Patients 1 to 9 underwent surgical alignment of the strabismus, four of them underwent BFT after the surgery. Patients 10, 11, and 12 were amblyopic with small angle strabismus under 5° without surgical indication, but they were included in the baseline analysis of the microperimetric parameters and one follow-up after three months without any intervention (non-treated).

**Table 1:** Clinical data of the patients

		Refraction		Preoperative BCDVA^a^		Postoperative BCDVA^a^	Surgical alignment of strabismus	BFT
N	Etiology	Treated eye	Fellow eye	Treated eye	Fellow eye	Treated eye		
1	S	plan − 0.50105°	plan − 0.50146°	0.7	0.0	0.7	Right	Right
2	SA	+ 3.00–4.50 10°	+ 0.50	1.5	0.0	1.4	Left	Left
3	SA	+ 4.25–1.25175°	+ 0.50–1.00160°	1.4	0.0	1.4	Right	Right
4	SA	+ 2.75–0.50 20°	−0.50 -1.00155°	0.3	0.0	0.3	Right	Right
5	S	plan	plan	0.0	0.0	0.0	left	–
6	S	+ 0.50–0.50180°	+ 1.00–0.50110°	0.1	0.1	0.1	right	–
7	S	−0.25 -0.50 10°	+ 0.75–0.75165°	0.0	0.0	0.0	left	–
8	SA	+ 1.25–2.75 70°	plan	0.2	0.0	0.2	Right	–
9	SA	+ 2.50–2.00 90°	plan	0.1	0.0	0.1	Right	–
10	SA	+ 3.50 + 0.50100°	plan	0.7	0.0	–	–	–
11	SA	+ 7.00	plan	0.7	0.0	–	–	–
12	SA	+ 2.25	+ 0.50–1.25 90°	1.3	0.0	–	–	–

*S* strabismus; *SA* strabismus and anisometropia; ^a^*BCDVA* best-corrected distance visual acuity (LogMAR) before biofeedback fixation training;

Inclusion criteria for all subjects were: absence of ophthalmological diseases (other than the diseases studied) and absence of neurological diseases or medications that could affect the central nervous system. Inclusion criteria for the group undergoing the training were surgical alignment of strabismus and amblyopia with decimal best-corrected distance visual acuity (BCDVA) of 0.5 in decimals (logMAR 0.3) or worse in the amblyopic eye. Moreover, subjects undergoing BFT had to agree of attending weekly training sessions and follow-up examinations in the eye clinic.

**Fig. 1:**
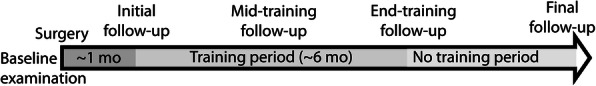
Complete protocol applied to the subjects undergoing surgical alignment of strabismus plus biofeedback fixation training

The study protocol included surgical alignment of the strabismus for patients with strabismic angle above 5°, follow-up examinations, and biofeedback fixation training (Fig. 1). For patients undergoing surgical alignment of the strabismus baseline and follow-up examinations were performed before and after surgery, respectively. Training subjects performed initial, mid-training, end-training, and post-training final follow-ups. Patients not receiving any treatment performed the baseline examination and one follow-up examination within three months of interval. The healthy volunteers of the control group were tested twice within one week interval.

#### Surgical alignment of the strabismus

Strabismus surgery was performed under general anaesthesia. In case of recession the muscle was reattached backwards at a targeted distance from the original insertion, using standard dosage patterns depending on the preoperative deviation [32, 33]. If resection was necessary, the muscle was shortened accordingly the standard Parks surgical tables [34]. There were no intraoperative or postoperative complications.

#### Clinical examinations

All subjects underwent a comprehensive ophthalmological examination including best corrected distance visual acuity (BCDVA), measured with electronic visual acuity testing charts in LogMAR values which allowed reordering optotypes randomly at each stimulus presentation after correcting refractive errors with spectacles and always by the same examiner. Moreover, orthoptic examination to measure horizontal and vertical deviations by cover test was performed. Some subjects were tested with near stereoacuity Stereo Fly test (LEA Test Intl, LLC, Chicago, USA) with circles test range between 800 and 40 arc sec. Furthermore, the intraocular pressure (IOP) measurement and fundus examination after dilatation of the pupils were performed.

The Macular Integrity Assessment microperimeter (MAIA; CenterVue, Padova, Italy) was used to evaluate macular sensitivity with the Expert Protocol (37 macular points up to 5° of eccentricity) using Goldmann standardized stimuli. The microperimeter is equipped with a scanning laser ophthalmoscope with real-time eye tracking system providing information about the fixation along the test. The patient’s task was, as in the conventional perimeters, to press a button in order to indicate the presence of a light spot whenever it was detected. The exported parameters, average threshold (average sensitivity of the macular points tested) and fixation stability were compared. Fixation stability is given as the bivariate contour ellipse area (BCEA) for 63 and 95% of the fixation positions and the frequency of fixation points (%) of the recorded points) within 1° (P1) and 2° (P2) of visual angle. Previous reports have shown details regarding the methods [18, 35] and the microperimetric parameters [19, 20].

#### Biofeedback fixation training

Based on previous reports [29, 30], biofeedback fixation training was performed monocularly to the amblyopic eye once per week for six months (with a mid-training follow-up examination) without interval. The biofeedback fixation training was identically delivered to all patients. Only the selection of the training retinal locus differed among the patients. The spontaneous preferred retinal locus (PRL) was selected as the training retinal locus for patients 1 and 4 with central fixation while the training retinal locus was another point near to the anatomical fovea (temporally shifted related to the original fixation area) for patients 2 and 3 with eccentric fixation, determined using high resolution OCT (SPECTRALIS system, Heidelberg. Engineering, Heidelberg, Germany).

During the training, the patient was requested to look at 1° white fixation spot monocularly (only the amblyopic eye) in a dark room. The examiner continuously guided the patient to inform him where to look while an auditory feedback beep increased its frequency as the patient approached fixation target by slowly moving the eyes. To guide the patient where to look, the words right, left, up, and down were used. Every time when the position of the eye reached exactly the desired fixation point, an auditory stimulus (Sonata Mozart) was delivered, indicating the correct position of the eye. The patient kept the fixation at the position as much as possible. Fixation stability was measured in all training sessions. Only patients participating to all training sessions and completing the six months period of training plus middle and final follow-up examinations were included in the study.

#### Statistics

The data analyzed are shown in the supplemental material. Group differences were evaluated using the non-parametric Mann-Whitney test (SPSS, Statistical Package for the Social Sciences, Hong Kong, China) *p*-values were corrected for multiple (six) comparisons. *p* values < 0.05 were considered statistically significant. Since only a small number of patients completed all protocol phases, we were careful with the statistical analysis for the paired comparisons. We described some results without drawing a statistical conclusion.

## Results

### Baseline macular sensitivity and variation of fixation stability

**Fig. 2:**
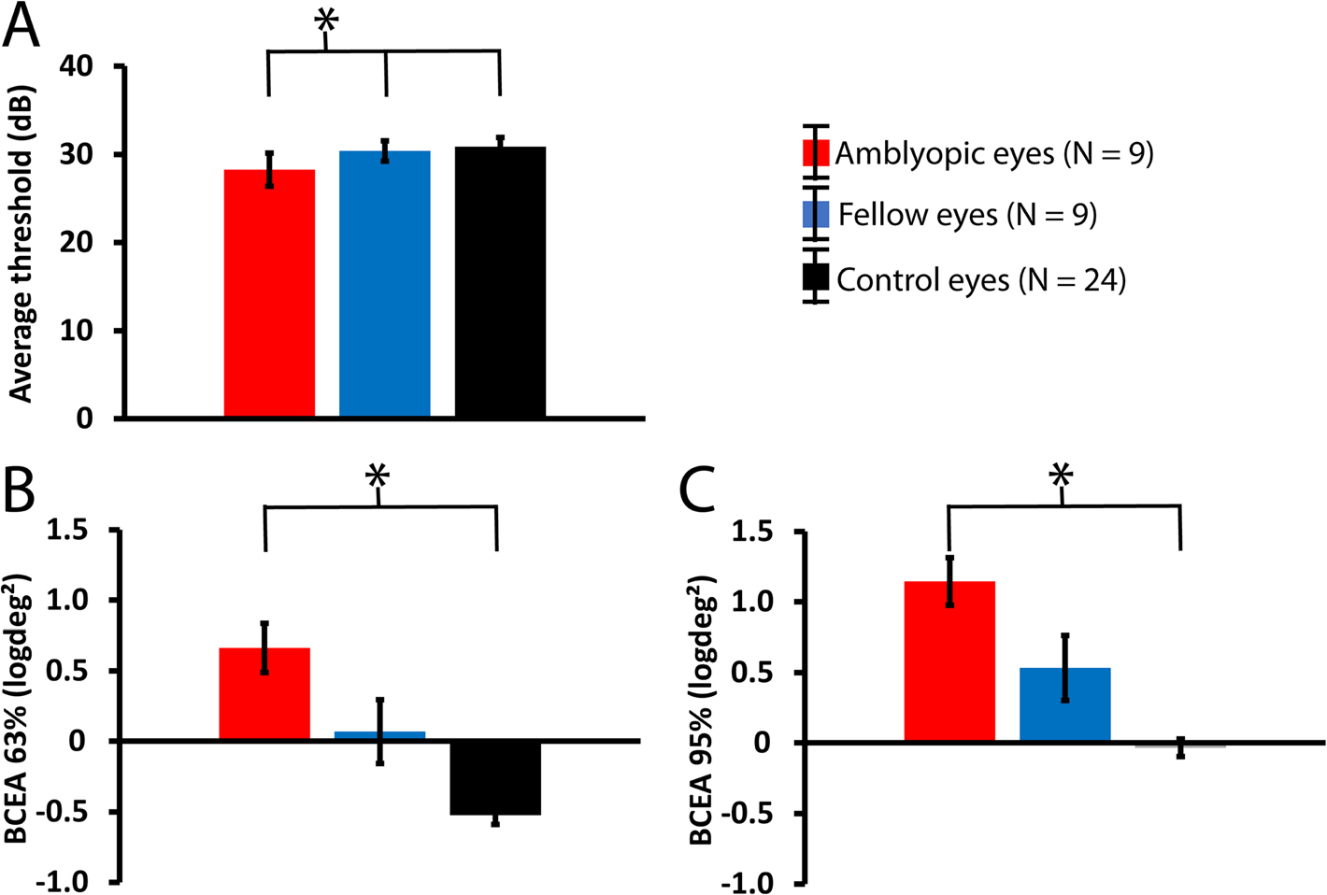
Mean (± one standard error of the mean) baseline microperimetric parameters. A: average thresholds (in dB), B: BCEA 63%, and C: BCEA 95% (logdeg^2^) for amblyopic (red; *N* = 9 eyes), fellow (blue; N = 9 eyes), and control (black; *N* = 24 eyes of 12 participants) eyes. All amblyopic patients (1 to 4 and 8 to 12 in Table 1) were included. Significant (*p* < 0.05) differences are marked with an asterisk

Baseline microperimetric parameters from nine amblyopic patients included in the study (subjects 1 to 4 and 8 to 12 in Table 1) and the healthy controls are shown in Fig. 2 for amblyopic (red; *N* = 9), fellow (blue; *N* = 9), and control (black; *N* = 24) eyes. As expected, macular thresholds (Fig. 2A) were significantly altered in the amblyopic eyes compared to both fellow (*p* = 0.024) and control (*p* < 0.001) eyes, but they were comparable in the fellow and control eyes (*p* = 0.290).

Fixation stability (Fig. 2B and C) expressed as BCEA (logdeg^2^) 63 and 95% index, respectively, was also significantly altered in amblyopic patients. A significantly increased BCEA in amblyopic eyes compared to control eyes (p < 0.001) was observed. Fixation stability was intermediate in the fellow eyes of the amblyopic patients as it showed statistically non-significant marginal difference (*p* = 0.066 and *p* = 0.084) compared to controls, however, statistically similar to amblyopic eyes (Bonferroni corrected *p* = 0.113).

**Fig. 3:**
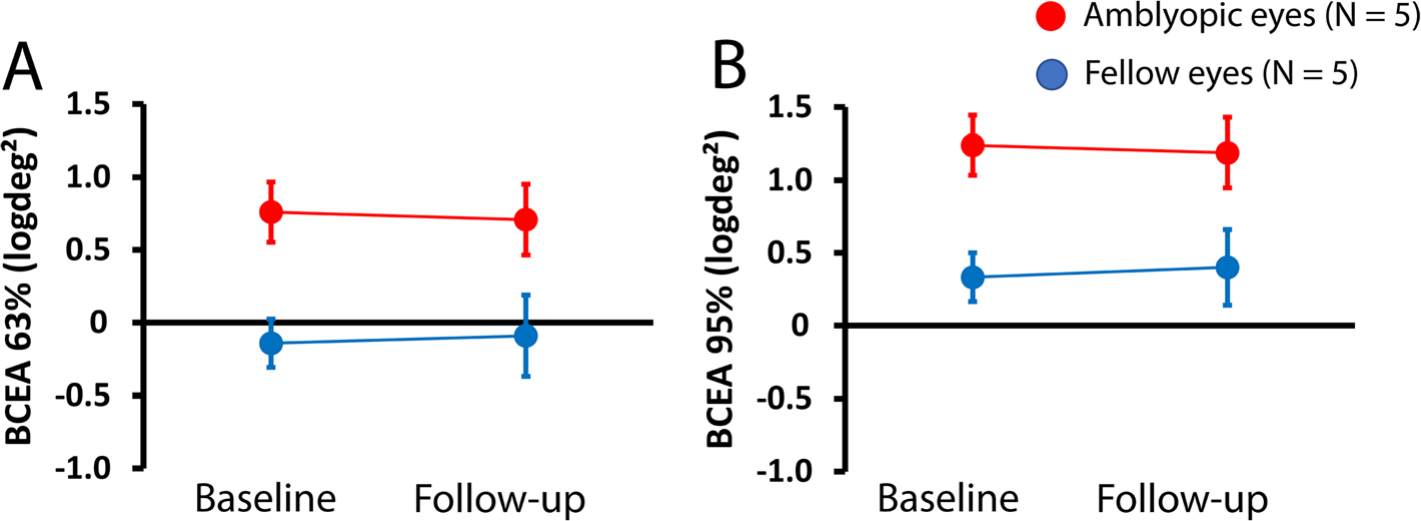
Mean (± one standard error of the mean) fixation stability obtained at the baseline examination and at the 3-month follow-up examination with no intervention between the two examinations. Amblyopic (red; *N* = 5) and fellow (blue; N = 5) eyes for BCEA 63% (**A**) and BCEA 95% (**B**) in logdeg^2^

We previously showed that fixation stability may improve after repeated microperimetric examination in patients with low vision due to age-related macular degeneration [36]. To check the variability of fixation stability in amblyopic and fellow eyes, a second examination was carried out in five amblyopic patients (subjects 3 and 4) before the training, and non-trained subjects 10, 11, and 12 in Table 1) three months after the baseline examination without interventions. Figure 3A and B show average fixation stability values of BCEA (63 and 95%, respectively) obtained during the baseline examination and the 3-month follow-up examination for amblyopic (red; *N* = 5) and fellow (blue, N = 5) eyes. Both fixation parameters, BCEA63% and BCEA95%, were relatively stable after three months.

### Effects of the surgery and surgery + BFT

**Fig. 4:**
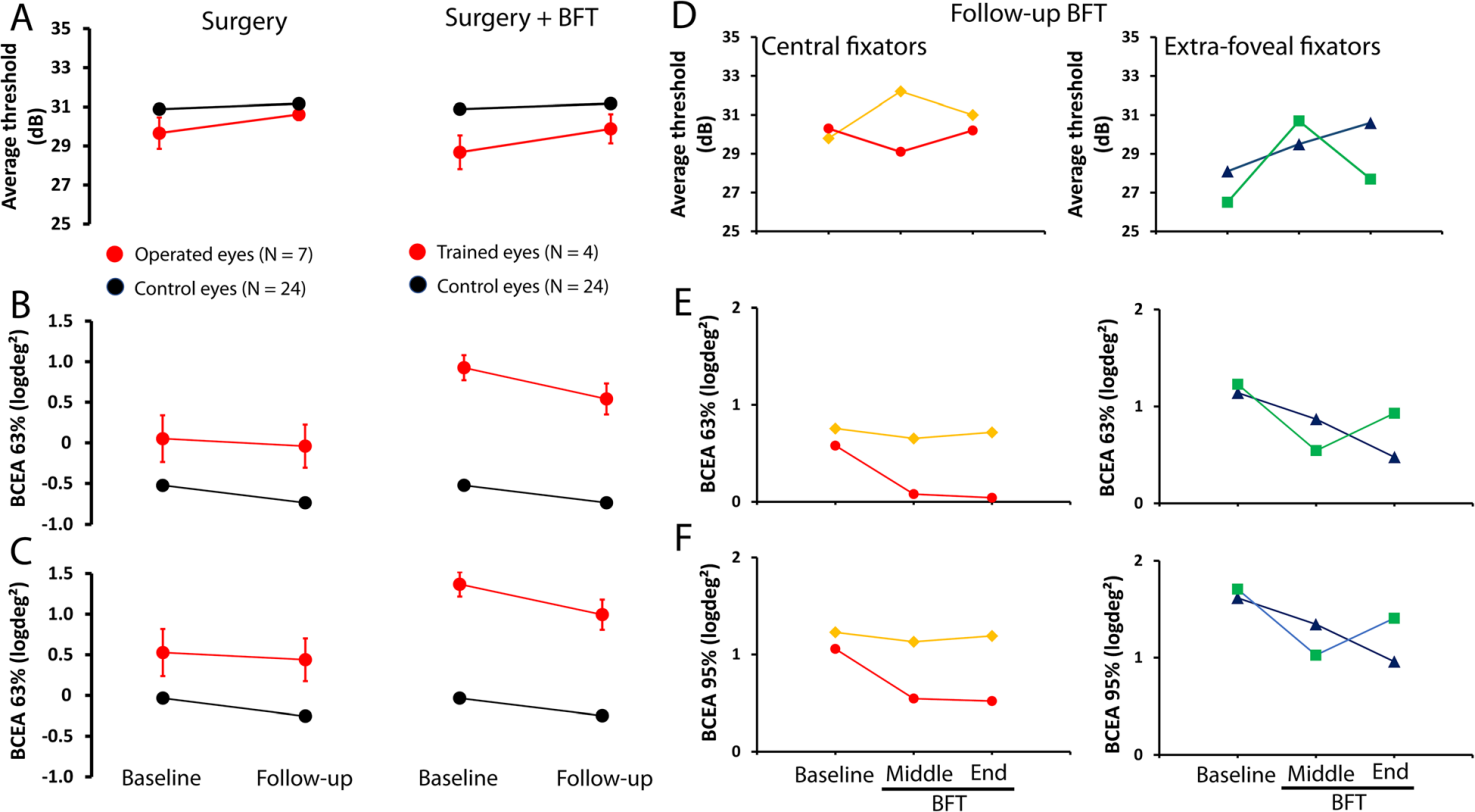
Effects of surgery and surgery + BFT on average threshold and fixation stability. Mean (± one standard error of the mean) of the average threshold (**A**), BCEA 63% (**B**), and BCEA 95% (**C**) for operated (*N* = 7; red symbols) eyes before and after surgery (first column) and before and after surgery + BFT (*N* = 4; second column). Control values are shown in black. Individual values obtained before BFT, in the middle of the training, and at the end of the BFT are shown for central (subjects 1 and 4) and extra-foveal (subjects 2 and 3) fixators for the average threshold (**D**), BCEA 63% (**E**), and BCEA 95% (**F**)

Examinations were performed before and about one month after the surgical alignment of the strabismus. Figure 4 (1st column) shows mean (± one standard error of the mean) of the microperimetric parameters before and after the surgery (red symbols) and control values (black symbols) obtained twice within one week interval. Average thresholds and fixation parameters, BCEA 63% and BCEA 95% displayed slightly changed values. Mean average threshold was 29.7 ± 0.8 (dB) before and 30.6 ± 0.3 dB after the surgery (Fig. 4A). The mean difference between the baseline and the follow-up examination was 0.9 dB, above the expected control variation that was 0.3 dB.

Fixation stability improved slightly after surgery. Before the surgery, the means were 0.05 ± 0.3 (logdeg^2^) and 0.53 ± 0.3 (logdeg^2^) for BCEA 63% and BCEA 95%, respectively. After the surgery, they were − 0.04 ± 0.3 (logdeg^2^) and 0.44 ± 0.3 (logdeg^2^) for BCEA 63% and BCEA 95%, respectively. However, the changes observed before (baseline) and after (follow-up) the surgery were not statistically significant (average threshold: *p* = 0.620 and fixation stability: *p* = 0.805.

Four patients (Patients 1 to 4 in Table 1) underwent BFT applied monocularly to the operated (amblyopic) eye during approximately six months. Figure 4 (2nd column) shows mean (± one standard error of the mean) of the microperimetric parameters before and after the surgery + BFT (red symbols) and control values (black symbols) obtained twice within one week interval. Slight increase of the mean average threshold (Fig. 4A) after surgery + BFT (average threshold before 28.7 ± 0.8 and after 29.9 ± 0.7 dB) was observed. Moreover, a prominent improvement in fixation stability was found. BCEA values (Figs. 4B and C) decreased (BCEA63% before 0.93 ± 0.2 and after 0.54 ± 0.2 logdeg^2^ and BCEA95% before 1.40 ± 0.2 and after 1.02 ± 0.2 logdeg^2^) at about half of the postoperative values after BFT. Figure 4D shows average threshold changes during BFT for the central fixators (subjects 1 and 4) and extra-foveal fixators (subjects 2 and 3). We observed that extra-foveal fixators were likely to drive the mean average threshold increase displayed in the mean plots (Fig. 4A), as the central fixators showed relatively similar average threshold before and after BFT. Interestingly, fixation stability (Figs. 4E e 4F) improved (decreased BCEA values) for all four subjects undergoing BFT.

**Fig. 5:**
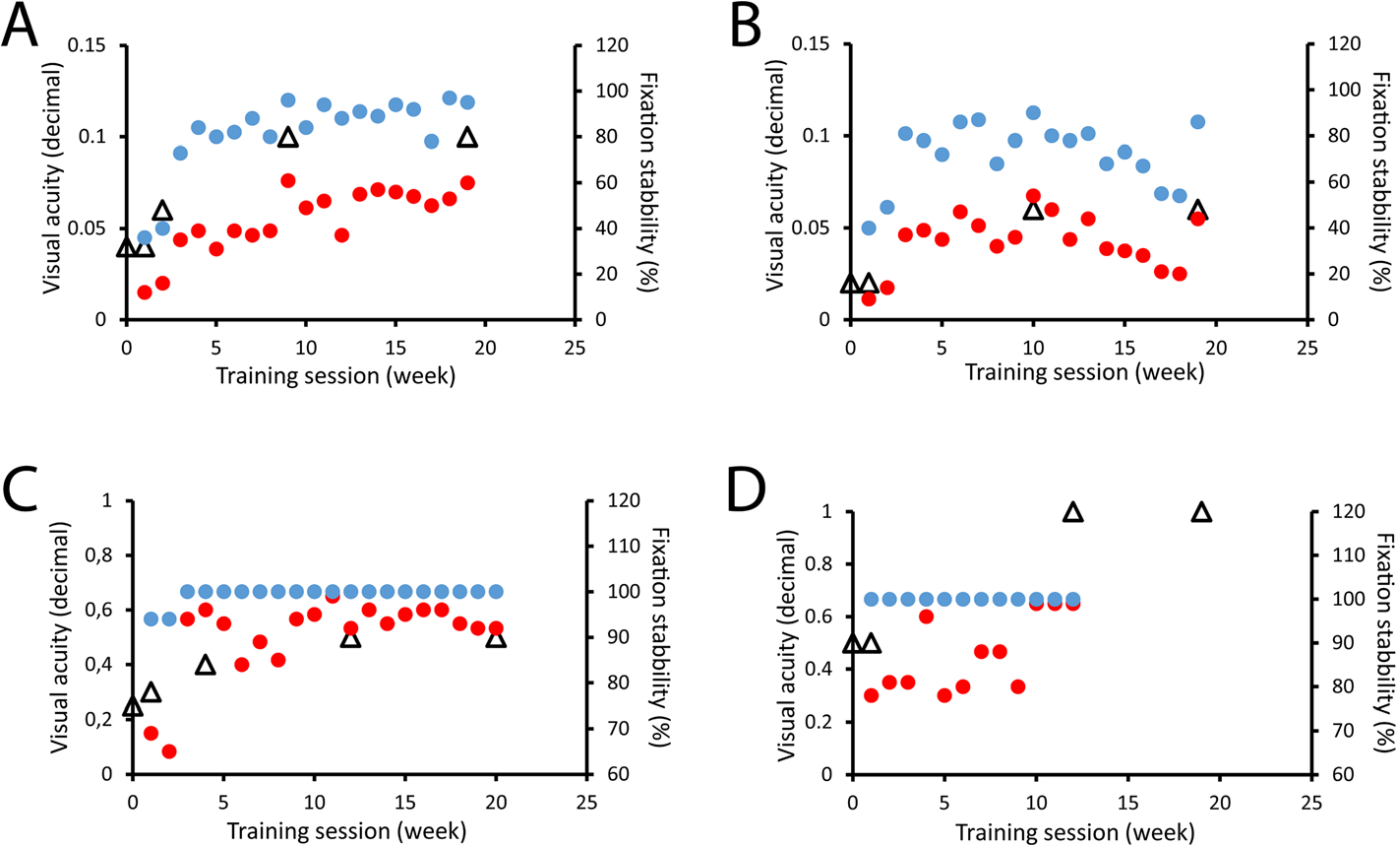
Biofeedback fixation training of four patients undergoing surgical alignment of the strabismus (Patients 1 = C, 2 = A, 3 = B, and 4 = D in Table 1). Fixation stability (P1 = red symbols and P2 = blue symbols) recorded during each training session. BCDVA (open triangles) examined during the training period for each of the four trained patients. y-axes at the left show BCDVA and at the right fixation stability. Fixation stability improved for all subjects. The training was interrupted earlier in one of the subjects (D) since 100% fixation stability was achieved with 1.0 BCDVA

Figure 5 shows fixation stability (P1 = red circles and P2 = blue circles) recorded during the training sessions and BCDVAs (open triangles) examined during the training period for each of the four patients undergoing BFT. Observe that y-axes of the visual acuity (left; shown in Snellen decimal here) are in the same range in Fig. 5A and B and in Fig. 5C and D to better show changes among the training sessions. BFT has been interrupted earlier in one of the subjects (Fig. 5D) since 100% fixation stability was achieved with 1.0 BCDVA.

The two subjects with central fixation, for whom spontaneous PRL was selected as the training point (Fig. 5C and D), more easily succeed in finding and keeping the eye position at the desired fixation point compared to the two extra-foveal fixators (Fig. 5A and B), for whom fixation points have been replaced. Accordingly, the central fixators achieved more stable fixation (P2 = 100% in Fig. 5C and P1 / P2 = 100% in Fig. 5D) what was not observed in the extra-foveal trained subjects. Along BFT and follow-up examinations there was no strabismus relapse or worsening of the postoperative angle stability.

## Discussion

The results of our present study show that: i) fixation stability is impaired in strabismic amblyopic adult eyes and ii) biofeedback fixation training (BFT) after alignment of the strabismus may improve fixation stability. Normal vision and appropriate binocular function depend upon the integrity of the eye’s movements to efficiently guide both eyes so that visual target is brought and maintained at the fixational (foveal) area (for review see Collewijn & Kowler, 2008) [37]. Accordingly, disturbed fixational eye movements may affect the quality of vision in subjects with strabismus and/or amblyopia [16, 38–42].

Fixational eye movements are characterized by slow oscillations of the eyes controlled by subcortical and cortical areas of the brain (for review see Kowler, 2011) [43]. The proper oculomotor control of the eyes ensures that the visual target is centrally placed on the fovea which is responsible for the best visual acuity that can be achieved by the visual system [44]. The present data emphasized [16, 38, 45, 46] that fixation in healthy eyes is quite stable while it is altered in patients with strabismus.

In subjects with normal vision, steady fixation allows keeping the visual target on the correct retinal position in both eyes so that visual attention is maintained until the object of interest can be detected or discriminated [47]. In contrast, unstable fixational eye movements continuously move the visual target out from the fixational locus, disturbing the attentional dedication to the visual target [39]. Here we show that fixation can be modulated in adult strabismic patients. We hypothesize that a more stable fixation could perhaps modulate monocular visual acuity and binocular vision in amblyopic eyes. Future investigations may address this question.

Amblyopia caused by strabismus, associated or not to anisometropia, may show eccentric and unstable fixation due to more severe extraocular motor alterations [40]. Previous reports taken together with the present data allow speculating that efforts to improve visual acuity of strabismic adult eyes with amblyopia should consider improving fixation stability in addition to stimulating visual perception of the amblyopic eye.

Although surgical correction of eye’s position in infants may successfully result in normal visual development during the critical period, reestablishment of fixation stability is not always achieved [16, 48]. In non-human adult primates, only a temporary improvement of fixation stability is observed after surgical treatment of strabismus [49]. Strabismus surgery delivered to the adult amblyopic eye is usually indicated as cosmetic correction, since visual perception and binocular vision are not expected to be changed after the surgical alignment. Functional improvements associated with surgical alignment of strabismus in adults are not clearly established. Some authors found that strabismus surgery significantly improves the self-reported quality of life due to a positive effect in visual performance [50, 51]. Pineles et al. found that binocular summation is improved after strabismus surgery [11]. However other authors showed that binocular function are not significantly modulated by the surgical alignment of the eyes when the critical period of development is over [52].

We hypothesize that improvements in fixation stability after correction of the eye position and BFT, may stimulate visual mechanisms responsible for the spatial vision in amblyopic eyes, increasing the possibility of visual acuity improvement. Among several types of behavioural rehabilitation for amblyopia [53–55], improvement of visual function provided by a short-term biofeedback training delivered to anisometropic amblyopic eyes of teenagers has been reported [30]. Here we reported that strabismic amblyopic eyes may benefit from BFT after surgical alignment of strabismus. Further studies with a large population may investigate the relations between clinical parameters, such as binocular balance [56], and visual improvements provided by BFT. In addition, to establish the proper criteria for selecting those patients who would more likely benefit from BFT for enhancing visual performance in amblyopic eyes.

Limitations of our study are the small sample size of participants. Further investigation with a larger population is necessary to confirm the present results. Moreover, although our results showed that surgical correction alone was not capable to improve fixation stability, a case-control study, in which some subjects are not enrolled in the training program and some subjects performing sham training after the surgical correction, would allow us to evaluate if the fixational improvements demonstrated here would be expected after surgical correction of strabismus or if they are related to the microperimetric biofeedback fixation training. Finally, we spculate that a more stable fixation of an amblyopic eye would help maintaining the primary position of the eye which could, in turn, reduce the possibility of strabismus relapse after the surgical correction.

## Conclusions

Biofeedback fixation training is a non-invasive, painless method and simple-to-perform therapeutic option for stabilizing fixation and improving visual function in several diseases affecting the central vision [22, 57]. It has been extensively reported to improve residual vision in patients with age-related macular degeneration and several other retinal conditions [58–61]. We hope the present report highlights the beneficial use of BFT as a therapeutic option to improve fixation stability in amblyopic eyes after surgical strabismus correction. Alternatively, this rehabilitation method could be associated with the standardized treatments as addictive therapy to improve visual function in adult amblyopic eyes as an attempt to enhance its effect.

## Supplementary Information

**Additional file 1.**

## Data Availability

The datasets generated and/or analysed during the current study are available in a supplementary excel file attached to the submission.
